# A New Application of Ether Vapour

**Published:** 1847-07

**Authors:** 


*A new application of Ether Vapour.*—It occurred to me lately, that the vapour of sulphuric ether might be used, instead of fumes of sulphur, in taking honeycomb from bee-hives. By experiment, I find that a very small quantity induces the full narcotic effect of the drug on these insects; the insensibility continues for nearly an hour, and is followed by complete recovery.

The humanity as well as the economy of the plan will, I think, recommend it; various simple means may be adopted for the application of the vapour; a proper precaution would be to envelope the hive with an air-tight hood, formed of some such material as oiled silk; the fumigation need not last longer than five minutes.—M. D.

*Ibid.*

